# A tactile perception method with flexible grating structural color

**DOI:** 10.1093/nsr/nwae413

**Published:** 2024-11-15

**Authors:** Yuze Qiu, Chunfei Yan, Yan Zhang, Shengxuan Yang, Xiang Yao, Fawen Ai, Jinjin Zheng, Shiwu Zhang, Xinge Yu, Erbao Dong

**Affiliations:** Key Laboratory of Precision and Intelligent Chemistry, University of Science and Technology of China, Hefei 230026, China; Institute of Humanoid Robots, Department of Precision Machinery and Precision Instrumentation, University of Science and Technology of China, Hefei 230026, China; Department of Biomedical Engineering, City University of Hong Kong, Hong Kong 999077, China; Institute of Humanoid Robots, Department of Precision Machinery and Precision Instrumentation, University of Science and Technology of China, Hefei 230026, China; Key Laboratory of Precision and Intelligent Chemistry, University of Science and Technology of China, Hefei 230026, China; Institute of Humanoid Robots, Department of Precision Machinery and Precision Instrumentation, University of Science and Technology of China, Hefei 230026, China; Institute of Humanoid Robots, Department of Precision Machinery and Precision Instrumentation, University of Science and Technology of China, Hefei 230026, China; Key Laboratory of Precision and Intelligent Chemistry, University of Science and Technology of China, Hefei 230026, China; Institute of Humanoid Robots, Department of Precision Machinery and Precision Instrumentation, University of Science and Technology of China, Hefei 230026, China; Institute of Humanoid Robots, Department of Precision Machinery and Precision Instrumentation, University of Science and Technology of China, Hefei 230026, China; Institute of Humanoid Robots, Department of Precision Machinery and Precision Instrumentation, University of Science and Technology of China, Hefei 230026, China; Institute of Humanoid Robots, Department of Precision Machinery and Precision Instrumentation, University of Science and Technology of China, Hefei 230026, China; Department of Biomedical Engineering, City University of Hong Kong, Hong Kong 999077, China; Key Laboratory of Precision and Intelligent Chemistry, University of Science and Technology of China, Hefei 230026, China; Institute of Humanoid Robots, Department of Precision Machinery and Precision Instrumentation, University of Science and Technology of China, Hefei 230026, China

**Keywords:** force and tactile sensing, structural color, vision-based tactile sensor, deep learning, wave optics

## Abstract

Affordable high-resolution cameras and state-of-the-art computer vision techniques have led to the emergence of various vision-based tactile sensors. However, current vision-based tactile sensors mainly depend on geometric optics or marker tracking for tactile assessments, resulting in limited performance. To solve this dilemma, we introduce optical interference patterns as the visual representation of tactile information for flexible tactile sensors. We propose a novel tactile perception method and its corresponding sensor, combining structural colors from flexible blazed gratings with deep learning. The richer structural colors and finer data processing foster the tactile estimation performance. The proposed sensor has an overall normal force magnitude accuracy of 6 mN, a planar resolution of 79 μm and a contact-depth resolution of 25 μm. This work presents a promising tactile method that combines wave optics, soft materials and machine learning. It performs well in tactile measurement, and can be expanded into multiple sensing fields.

## Teaser

This work introduces a novel tactile perception method that merges wave optics, soft materials, and machine learning, dramatically improving sensor tactile performance and ushering in new advancements in tactile technology.

## INTRODUCTION

Tactile perception is a good way for robots to explore and predict their environment [1] and is necessary for their various functions, such as shape perception and force feedback. Moreover, soft tactile sensors have good applicability and accuracy when robots face fragile targets in their work environment [2]. Thus, flexible tactile sensors with excellent performance are important for robots to perform complex tasks [3]. Tactile sensors have been extensively developed in recent decades using different conduction principles [4], which can be roughly classified into resistive [5], capacitive [6], magnetic [7], piezoelectric [8] and optical/vision-based types [9]. Compared with traditional tactile sensors, vision-based flexible tactile sensors have multiple advantages, such as high resolution [10], high stability, continuous tactile information, low cost and high signal-to-noise ratio [9]. Consequently, researchers have become increasingly interested in vision-based tactile sensors (VTSs).

Recently, several typical VTSs have been proposed and continuously developed. One of them, GelSight, proposed by Johnson and Adelson [11], is specialized for measuring the shape of contact areas. Via elastomeric gel deformation, it can capture the shape and texture of the object in contact and reconstruct a 3D height map of the in-contact surface using photometric stereo algorithms [12]. This scheme subsequently evolved into highly miniaturized tactile sensors with much higher resolution [3,13,14]. TacTip, proposed by Chorley *et al*. [15,16] in 2009, is an artificial fingertip-shaped tactile sensor that performs precise force-position recognition. The TacTip series has good integration capabilities and thus they can be used as standard devices in the field of soft machines [17]. Furthermore, a series of soft robot hands [18], soft machine feet [19] and soft machine systems [20] have been developed on the basis of TacTip.

However, relying solely on geometric optical information, such as illumination color changes with elastomer deformation, hampers the accurate force identification and localization of these VTSs, which poses a major limitation to their resolution and accuracy of force-position recognition. A marker array is an efficient method to overcome this limitation; by tracking the displacement of markers during elastomeric deformation, accurate assessments of the deformation field and the magnitude and position of the contact forces can be achieved. However, this method is greatly limited by the difficulties in producing smaller and denser markers. Furthermore, the limitation of the Rayleigh criterion is a dilemma hindering the development of the aforementioned method of dense marker arrays.

**Figure 1.**
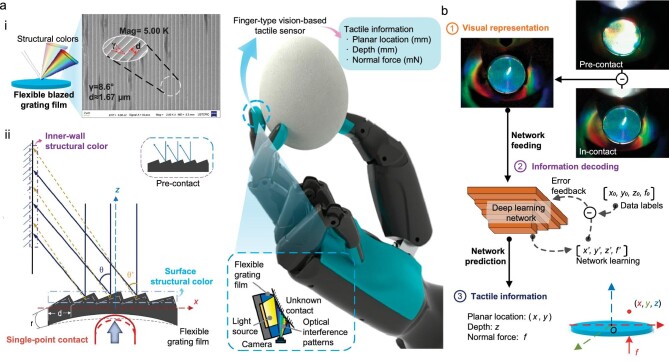
An overview of the tactile perception method with flexible grating structural color. The integration of optical interference, deep learning and soft materials endow the proposed method and its correlated sensor, IrisTact, with good performance and the potential for perceptual expansion. The conceptual diagram in the middle depicts the future application of a miniaturized IrisTact on robotic fingers for handling fragile objects like eggs and obtaining tactile information, highlighting the possibilities of this method afforded by relevant technological advancements. (a) Flexible blazed grating film: (i) by casting a soft, reproducible material onto a glass blazed grating, a flexible grating film is obtained, which possesses a surface periodic microstructure and unique structural colors (interference patterns); (ii) the schematic illustrates the generation of structural color features through interference effects of the flexible grating film. (b) Tactile estimation. When there is contact on the film of IrisTact, tactile information is visually represented by the film's structural color pattern. A deep-learning network is employed for automatic modeling and decoding of the tactile information derived from this structural color pattern. Ultimately, the network predicts and outputs the contact statuses, including the planar contact locations, contact depth and normal contact force.

Interestingly, when 3D markers are sufficiently dense and connected in rows or columns, their morphology converges to be like that of a grating, which has a periodic microgroove structure and is often used in optic experiments to produce specific diffraction interference phenomena. On the basis of the phase properties of light, the stripe patterns produced by the interference phenomena in wave optics can supply relatively high spatial resolution and information entropy. In terms of the development history of high-resolution sensors, many optical instruments or methods pursuing much higher precision and spatial resolution have shifted from using geometric optical information to using interferometric optical information. Examples include 4Pi-single-molecule switching super-resolution microscopy [21], synthetic aperture phase microscopy [22] and photonic-based nulling interferometry [23]. In line with these development trends, this study proposes a novel concept for a tactile perception method: we introduce optical interference patterns to serve as the visual representation for tactile information from sensors (Fig. 1), which allows the tactile estimation of much higher spatial resolution and accuracy by the sensors.

The blazed grating has high diffraction efficiency compared to other gratings. The surface of the blazed grating has periodic micron-serrated structures that can distinguish light rays with the same diffraction angles but different colors to produce physical structural colors based on diffraction-interference phenomena [24]. These physical structural colors, as a kind of high-resolution interference pattern, are ideal for use as the visual inputs of VTSs. Here, we fabricated a flexible grating film with optical structural colors using a glass reflective blazed grating (Fig. 1b), mimicking Morpho butterfly wings (Fig. S4). Utilizing multiple structural color features and deep-learning technology (Fig. 1b), we propose a VTS design based on this film, which has shown excellent performance in this work; considering its rainbow-like rich structural colors, it is named IrisTact. IrisTact not only achieves a higher comprehensive performance in terms of spatial resolution and force accuracy than concept prototypes [25–27] and representative state-of-the-art vision-based/optical tactile sensors (Table 1, see more comparisons in Section A.1), but also has high expandability, and with several simple changes or add-on parts it can easily complete different sensing tasks, like whisker sensing or vibration detection.

**Table 1.** Comparison between state-of-the-art tactile sensors and the proposed sensor design (IrisTact, highlighted in bold).

Sensor	Technology	Output	Area (mm²)	Spatial resolution (mm)	Force range (N)	Force accuracy (N)
Human finger [28,29]	-	Contact force, location, texture	-	1	40	0.06^a^
BioTac [30]	Fluid-based sensing	Contact force, location	484	2.4	10	0.26^b^
Gelsight-based [31]	Camera with soft elastomer	Tactile RGB image	252	0.030	22	1.9^b^
Geltip [32]	Camera with soft elastomer	Contact location	2513	5	-	-
GelSlim 2.0 [33]	Camera with markers and elastomer	Force map	1200	-	-	0.32^b^
GelSlim 3.0 [34]	Camera with markers and elastomer	Force map, contact shape	675	-	-	-
OmniTact [13]	Five cameras with elastomer	Contact location	3110	0.4	-	-
Soft-bubble [35]	Camera with inflated elastomer	Tactile depth image	-	-	-	-
Insight [10]	Camera with soft elastomer	3D force map	4800	0.4	2	0.03^c^
Tactip [17]	Camera with markers	Contact location	2500	0.2	-	-
DIGIT [14]	Camera with markers and elastomer	Contact shape	304	-	-	-
DenseTact 2.0 [36]	Camera with elastomer of randomized pattern	Tactile depth image, contact force and torque	1443.4	0.3633	3	0.41^d^
DelTact [37]	Camera with elastomer of dense random color pattern	Contact shape, contact force	675	-	9.67	0.3^b^
Kappassov*et al*. [38]	Camera, elastomer with color-coded POF^e^ inside	Contact normal force and location	1600	8	18	3.6^b^
ReSkin [39]	Magnetic field	Contact force and location	400	1	2.5	0.2^a^
Yan *et al*. [40]	Magnetic field	Contact force and location	∼400	0.06	-	0.01^b^
Yan *et al*. [41]	Magnetic field	Contact force and location	-	0.4	∼17	0.31^b^
GTac [42]	Magnetic field	Contact force and location	-	0.9	-	0.11^b^
Xie *et al*. [43]	Magnetic field	Contact force and location	∼95	4.8	15	0.012^a^
Epstein *et al*. [44]	Pressure	Contact force and location	184	∼1.11	25	0.65^b^
SaLoutos*et al*. [45]	Pressure, time-of-flight	Contact force, location and proximity	406	∼1.31	25	1.58^b^
9DTact [46]	Camera with soft elastomer	Contact shape, contact force and torque	432	0.0462	-	0.307^d^
Prototype [25]	Structural color of flexible grating	Contact normal force and location	∼491	∼1.11	2	∼0.006^d^
Smaller prototype [26]	Structural color of flexible grating	Contact normal force and location	∼314	∼1.0	2	∼0.15^d^
**IrisTact (this work)**	**Structural color of flexible grating**	**Contact normal force and location**	**∼491**	**∼0.079**	**∼1.02**	**∼0.006** ^d^

a Reported force accuracy as minimum distinguishable force. ^b^Reported force accuracy as root-mean-squared-error (RMSE) relative to an evaluation data set. ^c^Reported force accuracy as median error relative to an evaluation data set. ^d^Reported force accuracy as mean error relative to an evaluation data set. ^e^POF, plastic optical fiber.

The middle part of Fig. 1a also presents a future application vision for IrisTact: it will be integrated into robotic fingers, enabling rigid robotic hands to handle delicate and fragile objects, such as eggs or fruits, safely through the sensitive and rapid response of structural colors to tactile perception. Although the proposed sensor at present still needs to be minimized to fit into robotic fingers, this goal is feasible in principle, with further advancements and integration of miniature light sources and compact image capture technologies. Additionally, the impressive performance of IrisTact has proved that using optical interference patterns to provide a visual presentation of tactile information results in the enhanced performance of VTSs. Therefore, the proposed method not only has good research prospects but also heralds a new promising direction for the development of tactile perception systems with much higher force accuracy and spatial resolution, especially in the field of VTSs.

## RESULTS

### Principles of sensor designs

With regard to a previous concept prototype [25], the basic principle of IrisTact is explained briefly as follows: we etched and molded a blazed grating onto a flexible polydimethylsiloxane (PDMS) film via the transfer technique, and the first order of diffraction-interference patterns from the flexible grating film was captured by a camera via the vertical illumination of a parallel light source. This principle can be summarized using Equation (1):


(1) \begin{eqnarray*}d\left( {\sin \alpha + \sin \beta } \right) = m\lambda ,m = 0, \pm 1, \pm 2,...\end{eqnarray*}

where $\alpha $ is the angle between the incident light and the grating normal, $\beta $ is the angle between the diffracted light and the grating normal, *d* denotes the grating constant and *λ* denotes the wavelength of the m^th^-order diffracted light, where *m* is an integer indicating the order of diffracted light.

When an external load is applied, the flexible grating film deforms and causes changes in *α, β* and *d*, which leads to a special structural color change corresponding to the applied load. These high-resolution structural color changes are sensitive to tiny deformations [47], making them highly promising for applications in high-precision VTSs. Although the concept prototype [25] demonstrated the potential of this architecture for tactile sensors, its simple optical path design and large size (total length exceeding 250 mm) limit practical deployment, and its performance does not adequately match up with that of representative state-of-the-art sensors. For another smaller concept prototype [26], more comprehensive deep-learning pipelines were introduced into its data processing task. However, the use of a Raspberry Pi camera and an unstable artificial light source compromised the clarity of the structural color images. This issue was exacerbated by the significantly reduced size of the film, finally limiting its tactile estimation performance.

Recognizing these limitations, we undertook a comprehensive sensor redesign based on the proposed concept. While preserving the high spatial resolution optical structural colors of flexible grating, we reshaped and enhanced certain aspects of previous concept prototypes, such as optical representation, mechanical structure, and information decoding and tactile reconstruction. Detailed descriptions of these designs and improvements are provided below, and the fabrication and assembly of IrisTact are also showcased.

### Optical representation

The utilization of flexible grating structural colors is the foundational principle that previous concept prototypes and IrisTact use in order to realize tactile estimation. Previous work [25,26] focused on structural color on the surface of flexible grating films (hereafter referred to as surface structural color). Tactile force-position recognition is achieved through changes in surface structural color after a normal load is applied on the film. Nevertheless, surface structural color is a 2D planarized feature. Once it reaches a certain contact depth, the surface structural color converges to a colored line with few effective changes. This issue poses an obstacle to the amplitude recognition of normal loads, such normal load is a 3D space vector relative to the film plane. Therefore, a new spatial structural color feature must be identified to address this constraint.

Regarding the sensor, when the film is loaded via direct single-point contact and deforms, the flexible grating bulges toward the sensor interior, resulting in a loss of surface structural color. This loss increases as both the load and the degree of film deformation increase (Fig. S1). However, as the sensor's cavity is closed, these structural colors are actually reflected onto the sensor's inner wall, enabling their capture. Moreover, as the load and the deformation of film escalate, their reflection angle rises, thereby lowering their position on the inner wall. This phenomenon exhibits their strong correlation to the amplitude of the normal load applied to the film. Thus, it is practical to introduce the structural colors on the sensor's inner wall, hereafter referred to as inner-wall color, as a new spatial structural color characteristic for sensor data processing.

### Mechanical structure redesign

To improve the sensor's maximum working depth, we raised the ratio between PDMS and the crosslinking agent to 20 : 1 for much softer flexible grating films. We added an appropriate amount of metallic green powder into the PDMS solution to enhance the reflection strength of the inner-wall color. After experimental testing, the optimal proportion of PDMS to the crosslinking agent, black silicone pigments and metallic green powder was 220 : 11 : 2 : 8, which aims at a balanced performance between the surface structural color and the enhanced inner-wall color. As shown in Fig. S2a, the proposed film presents a stronger inner-wall color than the previous black film while the stability of the surface structural color is also ensured. Given the limited resolution of the Raspberry Pi camera and the large size of the industrial camera, a high-resolution camera module is used for tactile image acquisition. As the camera-shooting optical path and light-source optical path only have an angle of 19°, a semi-transparent mirror is used to fold the optical path. As shown in Fig. S2b, folding the optical path avoids structural interferences between the LED lamp bead and camera module while protecting acquired structural color images from disturbances related to the overlap of the optical paths mentioned above.

### Information decoding and tactile reconstruction

We employed a data-driven method of information decoding to directly and accurately predict and reconstruct single-point contacts from structural color image inputs using machine-learning techniques, namely, an adapted ResNet [48], which is a favored deep convolutional neural network architecture. The inputs to the model are preprocessed structural color images under contact, whereas the outputs are the planar coordinates of the contact on the flexible grating film, the contact depth and the normal contact force magnitude. To better solve the regression problem between images and numerical predictions, we deleted the network's SoftMax layer and enlarged its permissible input resolution. Then, to collect training and test data for the network, we used a 3D autonomous calibration platform [26] with a cylindrical indenter (diameter of 1.2 mm) to probe IrisTact. A force sensor (FUTEK LSB200) measured the ground truth of contact forces, with corresponding structural color images simultaneously recorded by the internal camera. To improve the performance of IrisTact, we performed improvements in its data set configurations:

- *Extended contact dwell time.* In the autonomous calibration process of image-data-set building, we extended the contact dwell time after each loading to reduce the error caused by the phenomenon in which the flexible film would release pressure while slowly rebounding after loading. After performing several tests, the flexible grating film reached a stable state at ∼10 s after loading.
- *Fusion of data sets.* We combined two kinds of data sets, points distributed symmetrically [25], and points with random cylindrical coordinates [26] (hereafter referred to as Fixed and Random, respectively). Regarding their performances, the Fixed data set excelled in predicting the load magnitude (mean error of ≈6 mN [25] and ≈150 mN [26]), illustrating the connection between structural color images and applied normal load changes. Conversely, the Random data set achieved higher accuracy in localizing the applied load (position error of ≈1.11 mm [25], and ≈1.0 mm [26]), highlighting the correlation between structural color images and load position variations. Therefore, by merging these two data sets, we created a more effective structural color image data set that more accurately and fully reflects the interplay of structural colors with both load locations and normal force magnitudes.
- *Dynamic difference method.* We implemented a dynamic difference method to enhance the utilization of structural colors more effectively. IrisTact exhibits a stable structural color pattern prior to contact, which can be captured as the pre-contact static reference image. The difference between this reference and the in-contact image is computed upon contact. This calculated difference subsequently serves as data for network training and recognition. Specifically, the dynamic difference method is applied selectively to the low-brightness inner-wall colors to highlight dynamic color changes while preserving the high-brightness surface structural color. The effect of this method is shown in Fig. 1b.

### Fabrication and assembly of IrisTact

**Figure 2.**
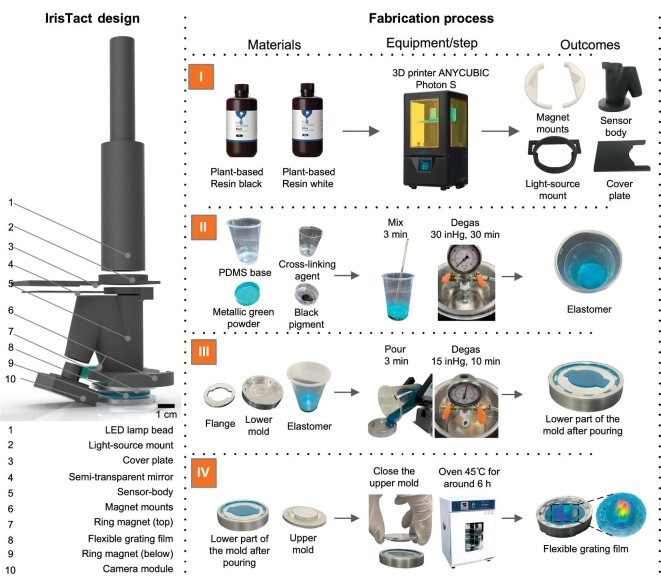
Fabrication process of IrisTact. Left: exploded view of IrisTact. Right: materials, processing steps and intermediate outcomes for the 3D-printed components and flexible grating film constructed from PDMS. ANYCUBIC owns the trademark and copyright of the names and pictures of ANYCUBIC Photon S and the black and white resin.

IrisTact is composed of 10 components, most of which, except the flexible grating film, were either 3D printed or off-shelf components. The complete fabrication process is shown in Fig. 2. Details of the fabrication process are presented in the Methods section.

### Performance

The performance of IrisTact was evaluated with respect to both static accuracy and dynamic recognition ability. The accuracy was measured via direct single-point contact estimation: the contact force of a single-point contact was localized, and its magnitude with corresponding contact depth was inferred. Then, we evaluated IrisTact's dynamic recognition ability via movement trajectory estimation for a single-point contact: the relative movement trajectory of a cylinder intender fixed on an optical platform over the sensor's sensing surface was inferred while the sensor was assembled on a robotic arm.

### Direct single-point contact estimation performance

**Figure 3.**
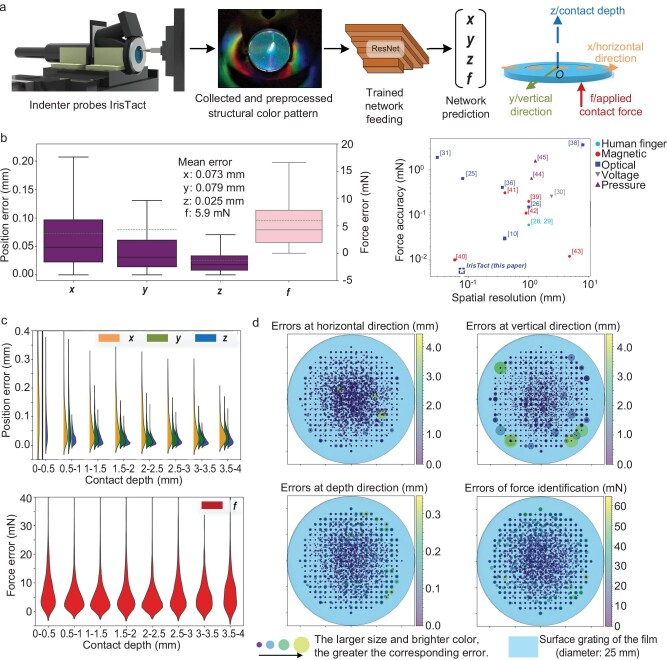
Direct single-point contact performance of IrisTact. (a) Estimation pipeline for inferring single-point contact position and force. The indenter fixed on the calibration platform probes IrisTact, and the structural color pattern is collected. After being preprocessed via the dynamic difference method, the image is fed into the machine-learning model, whose outputs represent the contact location (x, y and z for the planar coordinates and depth of the contact on the film) and normal contact force magnitude (f). (b) Overall performance of IrisTact (mean errors of x = ∼0.073 mm, y = ∼0.079 mm, z = ∼0.025 mm and f = ∼0.0059 N) and the comparison between state-of-the-art tactile sensors and IrisTact. (c) Statistical evaluation of the sensor's performance on the test data. Load localization and identification performances classified according to applied contact depth. The orange-, green- and blue-colored half-violins and red violins represent the distribution of prediction errors in x, y, z and f, respectively. (d) Spatial distributions, projected onto the surface grating, of quantification errors of the force localization and identification for the same test data. The larger the size of the dot and the brighter its color, the greater its corresponding error.

We evaluated the sensor's direct single-point contact recognition accuracy in terms of localization and force sensing for an applied normal load magnitude up to ∼1.02 N (Fig. 3a). All numerical results are for the points in the test data set, which do not appear in the training data set. The overall mean (planar) localization precision of IrisTact is better than 0.1 mm. In particular, the precision values are ∼0.073 mm in the horizontal direction (x) and ∼0.079 mm in the vertical direction (y), and the overall mean prediction precision is ∼0.025 mm in the contact depth (z). The normal load magnitude precision is ∼0.0059 N. The displacement platform has a repeatability of 2 μm, and the force sensor has a non-linearity of 0.1% of rated output. The accuracy of IrisTact in terms of force magnitude is remarkably stable across different applied contact depth ranges, but the errors in spatial localization (contact depth, and horizontal and vertical directions) slightly increase when the applied contact depth is in the range of 0–0.5 mm (Fig. 3c). For shallow contact depths (<0.5 mm), the spatial localization accuracy deteriorates, presumably because the elasticity of the PDMS material inhibits the film's sensitivity to small deformations, making it difficult for its structural color images to derive clear differences with the static status at small deformations. For much deeper contact depths exceeding 3.5 mm, the error in the force amplitude similarly increases. This phenomenon may be attributed to the inner-wall color losing effectiveness, which converges from the periphery to the surface grating when positioned near the maximum depth (4 mm). The larger the contact depth is, the closer the inner-wall color is to the surface grating. Thus, the difference between the inner-wall color at different depths (between 3.5 and 4 mm) decreases; at this time, the surface structural color has shrunk to a color line with negligible difference (Fig. S1b), causing loss of spatial characteristic information. These factors jointly lead to an obvious increase in load identification errors at a contact depth of >3.5 mm. Another factor may also have contributed to the result. In producing the Random data set, we used a 1D normal distribution to randomly generate contact depths for contact points, which has a mean of 2 mm and a variance of 2/3 mm. This approach resulted in a smaller amount of data for the contact depths in these two intervals (0–0.5 mm and 3.5–4 mm) in the overall data set compared with the other depths. This scenario may have also led to poor recognition performance of the network at the aforementioned two contact depth intervals. As the deformation of the flexible grating film is obviously positively correlated with the magnitude of the interaction force, the performance of IrisTact across different force ranges is similar to its performance across the corresponding applied contact depth ranges, as presented in Fig. S8a.

Furthermore, as the load position approaches the edge of the working surface, the non-linearity of the flexible grating film between the applied force and film deformation increases [25], and the correlation between the changes in structural colors and film deformation is substantially weakened. This factor eventually leads to an increase in the errors of force identification and localization, especially in the vertical direction, when the load position moves closer to the edge of the flexible grating film at a radius between 7.5 and 10 mm (Fig. S8b). We evaluated IrisTact's accuracy in localizing the test points on the flexible grating film (Fig. 3d). The accuracy is stable and good (smaller and darker dots, Fig. 3d) inside the circular area with a 7.5 mm radius from the center of the film. More noticeable errors (larger and brighter dots, Fig. 3d) appear near the edge of the film.

### Dynamic-contact-trajectory estimation performance

**Figure 4.**
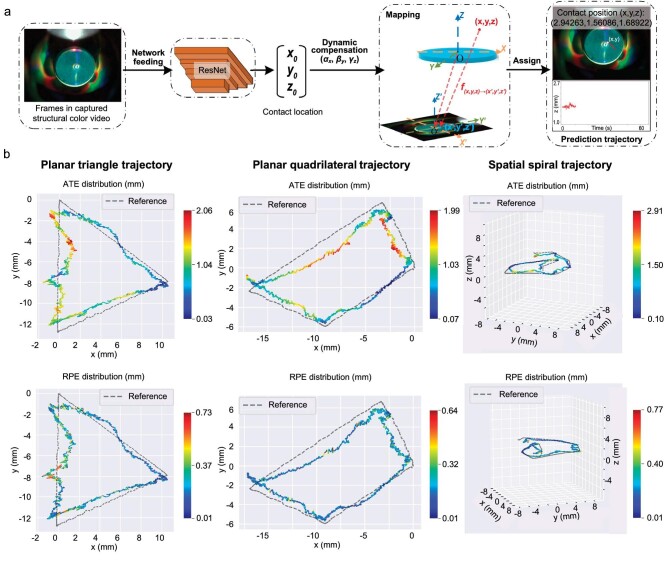
Evaluation of IrisTact's dynamic recognition of contact trajectory. (a) Pipeline for recognizing the dynamic trajectory. ResNet is used to predict the contact location for each frame of the recorded structural color video. After dynamic compensation, these locations are mapped and displayed on the respective frames to visually demonstrate the movement of contact points over time. (b) Graphical evaluation and comparison of IrisTact's dynamic recognition performance, across different trajectories, with the reference trajectory captured by the motion capture system.

We used a UR-5 robotic arm to test the dynamic recognition ability of IrisTact for moving contacts on its sensing surface. In this experiment, the flexible grating film was maintained in contact with an indenter fixed underneath on an optical platform. The movement of the manipulator caused a relative sliding of the indenter to the film (Fig. S9). To confirm the real trajectory of the terminal of the robotic arm where the sensor was mounted, we used a commercially available optoelectronic motion capture system to track and record the trajectory. The marker was a fluorescent ball fixed onto IrisTact and served as the tracking target. During the acquisition of the static training and test data sets, the rebound phenomenon caused by the lack of a rigid skeleton and the elasticity for the flexible grating film presented problems that could be solved by extending a certain dwell time after each contact. However, this solution is unrealistic for dynamic tactile data, such as videos. Thus, we introduced three empirical coefficients for the dynamic compensation of the sensor's output to reduce the effect of this phenomenon (Fig. 4a). These empirical dynamic compensation coefficients were estimated on the basis of the scale correction parameters obtained from multiple Sim (3) Umeyama alignments between the IrisTact results and motion capture system results, in which multiple standard trajectories were tested. The prediction output of the sensor was amplified appropriately. The graphical evaluation and comparison of the predicted trajectory with the real trajectory recorded by the motion capture system for the dynamic recognition performance of IrisTact are shown in Fig. 4b.

Here, absolute trajectory errors and relative pose errors (ATEs and RPEs, as defined in Sturm *et al*. [49]) were introduced to comprehensively and accurately assess IrisTact's prediction results. For the planar trajectories with simple shapes (triangle and quadrilateral/four-sided configurations), the ATEs between the prediction trajectory of the sensor and the real trajectory were lower than 2.1 mm, with mean and median values of <1 mm. These results show the good precision of IrisTact in dynamic trajectory prediction. Meanwhile, the RPEs of the prediction trajectories were stable at <1 mm, with mean and median values of ∼0.2 mm, demonstrating the high local accuracy and low trajectory drift of IrisTact's prediction results. For the highly complex spatial spiral trajectory, IrisTact also presented a low ATE (mean and median of ∼1 mm) and RPE (mean and median of <0.2 mm), although their maximum values slightly increased. Moreover, although a certain gap exists between IrisTact's prediction trajectories and the real trajectories in their shapes, the sensor still has a good dynamic response in all three directions (horizontal (x), vertical (y) and contact depth (z) directions), with small phase differences between its prediction and real trajectory, as shown in Fig. S13. This experiment not only demonstrated the good performance of IrisTact for both dynamic contact trajectory recognition (i.e. for both 2D planar sliding and 3D spatial sliding contacts) and dynamic response but also proved its great potential in high-precision and low-latency contact motion prediction. These lay a solid foundation for the future application of IrisTact as a tactile sensing unit in robotic hands, enabling precise manipulation tasks such as object sliding and grasping.

### Sensing expansions of the IrisTact sensor method

By combining wave optics interferences, soft materials and machine learning, the proposed sensor method achieves excellent force localization and identification, as confirmed by the good tactile sensing capabilities of IrisTact in both static and dynamic scenarios. Moreover, the sensor's modular design facilitates the easy adaptation of the proposed tactile perception method to a wide range of sensing applications, accommodating different shapes and functional requirements. The rich information inherent in structural colors and their sensitive response to changes in tactile status also endow our method with good flexibility: it is not limited to data processing through deep learning; appropriate image processing procedures can also meet its needs in information extraction and presentation among various sensing applications. Here, we provide simple examples of how our sensor method can be extended to various sensing applications.

### Low-frequency vibration sensing

As previously discussed, the structural color pattern of IrisTact closely correlates with the planar position and the contact depth of a single-point contact. The film's elasticity enables it to respond quickly to various contacts, allowing for the vivid depiction of changes in contact status through alterations in the structural color patterns, without focusing on accurate force localization and recognition. This feature is highlighted in Video S3, where the structural color patterns of IrisTact sensitively respond to the subtle and low-frequency vibrations of an UR-5 robotic arm that are nearly invisible to the naked eye. This allows for the effective monitoring and analysis of small external vibrations transmitted to the flexible grating film of IrisTact.

**Figure 5.**
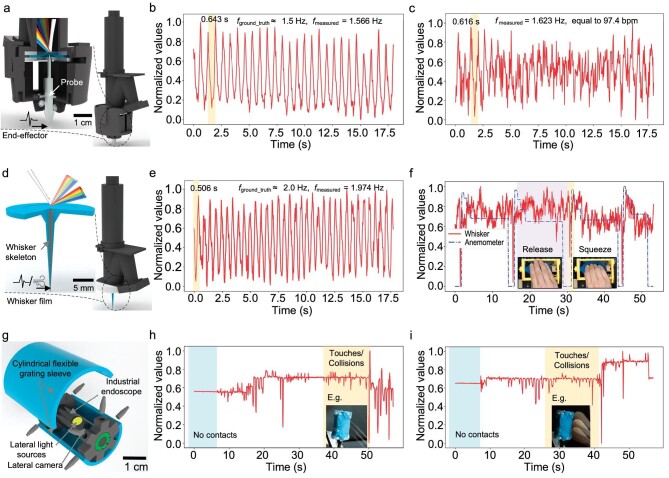
Perceptual extension examples of the IrisTact tactile perception method. (a–c) Equipped with an end-effector, the sensor prototype is adept at sensing external low-frequency vibrations, which induce correlated structural color variations. It captures low-frequency vibration signals generated during consistent sliding activities (b), and measures the pulse rate at a human wrist (c). (d–f) Incorporating a whisker on the flexible grating film enhances the film's responsiveness to subtle external disturbances. The adaptation of this whisker film enables IrisTact to detect external disturbances, such as low-frequency vibrations occurring during stable sliding activities (e) or the passage of airflow (f) in turn. In the right graph, blue lines represent reference measurements taken by an anemometer, while red lines denote results from the whisker sensor. (g–i) Outfitted with a cylindrical flexible grating sleeve, this endoscope is capable of detecting 3D contacts with a mild iron wire (h) or a cosmetic brush (i). The strong variations in structural color patterns triggered by these contacts are recorded by its lateral cameras. The frequency and amplitude of these variations, visible in the video, provide detailed information about the nature of 3D contacts, such as intensity and location of these touches or collisions.

Here, an additional end-effector is attached to the working side of IrisTact, enabling the direct transmission of external vibrations to the pretensioned flexible grating film through its central probe (Fig. 5a). Upon initiation of vibrations, the structural color variations recorded in the video are magnified by the Eulerian video magnification method [50], and then the main frequency of these external vibrations is deduced (Fig. S33). This vibration sensor prototype is capable of measuring low-frequency vibrations, such as pulse-like signals with frequencies ∼1.5 Hz generated by relative sliding at a certain speed between the external probe and the scales of a steel ruler (Fig. 5b). Moreover, it is also feasible to detect the pulse at a person's wrist and measure the frequency (Fig. 5c). All curves of the extracted vibration signals were calculated according to the weighted brightness value for each video frame.

### Whisker sensing

Thanks to the freedom of custom production in creating a flexible grating film, the film can incorporate its own probe instead of add-on components to actively sense external disturbances. Using integrated casting molding, a rigid-flexible coupling whisker, with a soft covering and a rigid internal skeleton, can be constructed on the working side of the flexible grating film (Fig. 5d). The whisker's structure (i.e. skeleton length) can be freely modified to suit specific usage scenarios. When a disturbance meets the whisker, jittering occurs on the whisker and is then transmitted into the film, causing corresponding structural color variations. Based on these variations, the disturbance information can be easily inferred. Here, an example of the whisker sensor prototype with a full-length skeleton is supplied. This whisker sensor prototype can not only measure the pulse frequency produced by the relative sliding between the whisker (Fig. 5e) and a plastic ruler's scales but can also perceive disturbances brought by passing artificial airflows (Fig. 5f). Its data processing pipeline is similar to that of the above-mentioned vibration sensor prototype.

### 3D contact perception

In addition to the use of the entire IrisTact as a basis for the expansion of different perceptions, the flexible grating film can be solely modified and applied to a variety of different perception tasks owing to the flexibility of its design and production. Here, we implemented our sensor method into 3D contact perception for endoscope systems (Fig. 5g), using an industrial endoscope as an example.

By changing the film's mold into a rectangular shape, rectangular flexible grating films can be obtained to fit most mechanical structures [51]. A cylindrical flexible grating sleeve is obtained by appropriate bending and fixing of rectangular flexible grating films. With custom fixings, this sleeve can be mounted on an industrial endoscope that has two sets of lateral lenses and lateral light sources. When an object touches/collides with the flexible grating sleeve, the structural color patterns change, resulting in obvious structural color variations captured by the lateral lenses. These variations, when recorded in a video, form sharp jumps in the time sequence of the normalized brightness value of video frames. Thus, by monitoring these jumps, this endoscopic system can perceive contacts with surrounding objects or environments, such as a mild iron wire (Fig. 5h) or a cosmetic brush (Fig. 5i). Furthermore, the intensity of these contacts is indicated by the magnitude of jumps in the normalized brightness values. This prototype system offers a promising approach for safe and lower-irritation collision perception when using medical endoscopes inside the human body, although its size, especially the film thickness, needs to be reduced in subsequent studies to further meet the requirements for medical use.

## DISCUSSION

In this work, we presented an improved VTS named IrisTact that uses structural colors from a flexible grating film to localize and identify applied normal loads and contact depths on its sensing surface. The sensor has an overall normal force magnitude accuracy of ∼6 mN, a planar localization accuracy of ∼79 μm, and a contact depth prediction accuracy of ∼25 μm. This sensor can independently infer the locations and magnitudes of normal loads, including contact depths within 4 mm. Owing to the multiple high-resolution structural color features utilized, IrisTact can distinguish small contact forces (6 mN) and detect tiny distances (79 μm), surpassing recent state-of-the-art VTSs in terms of both force localization and identification. A detailed comparison between IrisTact and other tactile sensors is shown in Table 1 and Table S1. Moreover, IrisTact has good precision and dynamic responsiveness to dynamic contacts on its working surface, and its dynamic recognition accuracy can be greatly improved with further precise dynamic calibration.

In contrast to traditional tactile sensors, the majority of VTSs detect deformations and compute interaction forces by combining the extrusion deformations of elastomers with illumination color intensity changes or marker tracking. The key to these methods of tactile detection is the measurement and calibration of elastomer deformations, whose effect is closely related to reflection effects and homogeneous lighting. These elements, coupled with the inevitable inhomogeneity of experimentally produced elastomers, often become more complicated with the adoption of linear elasticity theory and optical methods, if the aim is to properly model these elastomers theoretically. This scenario is also true for our flexible grating films, which combine wave optics with soft materials. To solve this dilemma, we adopted a data-driven approach and used end-to-end learning, allowing all structural color effects of IrisTact to be modeled automatically. A disadvantage of using our approach is that a precise multi-axis calibration platform is needed to collect large amounts of reference and test data; however, once the platform is constructed, it can not only automatically obtain and calibrate all the data but also be used to collect data for the different contact geometries and for the sensor extensions based on IrisTact. Furthermore, only the calibration of the initial sensor positions on the platform and the design of corresponding supports are needed.

Here we mainly demonstrated the performance of IrisTact in both static and dynamic scenarios of direct single-point contacts with a 1.2 mm-diameter cylinder indenter. The multipoint contact situation was discussed in the previous work of the concept prototype, illustrating the feasibility of our tactile perception method in discerning multipoint contacts. This topic will be further explored in our future work on IrisTact. Similarly, knowing how to apply IrisTact to recognize complex contact morphologies needs further research. Next, we plan to combine a dense marker array with structural colors to provide IrisTact with a much richer tactile perception of the contact surface geometry. Of course, we must acknowledge that the current excessive height of IrisTact limits its use in various situations, even though this was a compromise made to ensure the stability and richness of the structural colors, considering that obviously a parallel light source (an LED lamp bead used here) can better display the structural colors of the flexible grating. However, the minimization of IrisTact into a fingertip tactile unit shown in Fig. 1 is still feasible in principle and technology with further advancements and integration of miniature light sources and compact image capture technologies. In the future, we will also test the effects of different light sources on structural colors to determine the appropriate light source selection for the miniaturized IrisTact. Furthermore, the architecture of IrisTact still provides guidance for the design of subsequent miniaturized structures. Also, the mechanical structure, which uses stable mechanical fixations for module mounting, is important in subsequent designs; this aspect is limited in the current design.

Moreover, due to the 1D blazed grating used as the master grating for our flexible grating film, the structural color patterns inevitably exhibit dependence on light incidence and polarization. In this work, strict requirements are imposed on the optical system of the sensor to resist effects from these dependances (as shown in Fig. S2), including limiting the optical path by enclosing the sensor chamber, fixing the flexible grating film after installation, and adapting a parallel light source with stable color temperature. In contrast to 1D gratings, 2D gratings and ingeniously designed 2D concave structures perform well at eliminating directional dependence. In subsequent work, we plan to integrate IrisTact with 2D gratings and 2D concave structures to reduce the proposed tactile perception method's dependency on incident light and optical paths.

In general, our tactile perception method offers a promising direction for manufacturing high-precision VTSs, namely VTSs through the utilization of interference patterns like structural colors from flexible gratings. Combining machine learning, soft materials and optical interferences means this method performs very well with respect to force localization and identification, as confirmed by IrisTact. Moreover, our sensor method can be easily extended to multiple sensing fields, aiming at various robot body parts with different shapes and needs.

## METHODS

### Sensor structure and camera type

A semi-transparent mirror was used to fold the sensor's internal optical path. The right side of the sensor body (Fig. S2) consisted of two similar cylindrical structures that follow the illumination light path and the reflected light path generated by the flexible grating film, positioned at an angle of 19° between each other. The left part of the sensor body was shaped like a cone to fit the size and field of view (FOV) of the lens of the camera module. IrisTact had a circular sensing surface with a diameter of 25 mm, namely the surface grating size of the flexible grating film. The camera module (SY011HD, Shenzhen VISHINSGAE Co., Ltd.) used had a resolution of 1920 × 1080 and an FOV of 44°.

### Light source

We used an LED lamp bead (XPeBWT-L1-0000-00G51) as the light source, with a color temperature of 6200 K. The spot diameter of the LED lamp bead was 28 mm. The white light emitted from the LED lamp bead was collimated by a set of lenses inside it. The forms of the light source of IrisTact were no-limited, although a lamp bead with collimators is recommended in this work. The main requirements for the light source used in IrisTact are the color temperature and collimation. The flexible grating film reacted differently to lights with various color temperatures, embodied in the tonal variation of both no-contact and in-contact structural colors. The collimation of the light source affected the illumination of the surface grating and accuracy of the structural colors; both aspects needed to be ensured with respect to the size of the sensor. The size of the light source spot should fully cover the range of the surface grating of the flexible grating film.

### Flexible grating films and sensor fabrication

The flexible grating film was completed by casting it with PDMS (Dow Corning SYLGARD™ 184 Silicone Elastomer), which was obtained by mixing a special ratio of 220 : 11 : 2 : 8 (the process of ratio confirmation is shown in Data A.2). After degassing in a vacuum chamber, the prepared PDMS was poured into a lower mold. After another short degassing period, the upper mold with a circular blazed grating (600 lines/mm; blazing angle of 8.6°) was closed, and the entire mold was placed in an incubator for 6 h at 45°C. After curing and demolding, the flexible grating film was fixed using two ring magnets and assembled on the working end of IrisTact. Owing to the modular production and installation method of the tactile section, the flexible grating film could be easily replaced, and the orientation of the surface grating could be adjusted. However, we did not evaluate the quality of the results obtained without retraining.

The whole mold for film production was made of aluminum alloy and customized by computer numerical control machining. All the 3D-printed components were printed by a 3D printer (ANYCUBIC Photon S; black and white resin as the material; note: ANYCUBIC owns the trademark and copyright of the names and pictures used in Fig. 2). The incubator model was DH-250AS, which is an electric temperature box; the trademark and copyright of these names and photographs are owned by Beijing Kewei Yongxing Instrument Co., Ltd.

## Supplementary Material

nwae413_Supplemental_File
