# Supplementary material for: A tactile perception method with flexible grating structural color
Source: Natl Sci Rev. 2024 Nov 15;12(1):nwae413. doi: 10.1093/nsr/nwae413 (PMC11702659; doi:10.1093/nsr/nwae413)
Supplement: nwae413_Supplemental_File [file nwae413_supplemental_file.zip › Teaser text.docx]

**Teaser text**

This work introduces a novel tactile perception method that merges wave optics, soft materials, and machine learning, dramatically improving sensor tactile performance and ushering in new advancements in tactile technology.
